# Analysis of equilibrium binding of an orthosteric tracer and two allosteric modulators

**DOI:** 10.1371/journal.pone.0214255

**Published:** 2019-03-27

**Authors:** Jan Jakubík, Alena Randáková, Esam E. El-Fakahany, Vladimír Doležal

**Affiliations:** 1 Department of Neurochemistry, Institute of Physiology CAS, Prague, Czech Republic; 2 Department of Experimental and Clinical Pharmacology, University of Minnesota College of Pharmacy, Minneapolis, MN, United States of America; Danish Cancer Society Research Center, DENMARK

## Abstract

Allosteric ligands bind to receptors at sites that are distinct from those endogenous agonists and orthosteric pharmacological agents interact with. Both an allosteric and orthosteric ligand bind simultaneously to the receptor to form a ternary complex, where each ligand influences binding affinity of the other to the receptor, either positively or negatively. Allosteric modulators are an intensively studied group of receptor ligands because of their potentially greater selectivity over orthosteric ligands, with the possibility of fine tuning of the effects of endogenous neurotransmitters and hormones. The affinity of an unlabelled allosteric ligand is commonly estimated by measuring its effects on binding of a radio-labelled orthosteric tracer. This scenario is complicated by many folds when one studies the kinetics of interactions of two allosteric agents, added simultaneously, on binding of an orthosteric tracer. In this paper, we provide, for the first time, theoretical basis for analysis of such complex interactions. We have expanded our analysis to include the possibility of having two allosteric modulators interact with the same or different sites on the receptor. An added value of our analysis is to provide a tool to distinguish between the two situations. Finally, we also modelled binding of two molecules of one allosteric modulator to one receptor.

## Introduction

Pharmacological ligands can be divided into orthosteric and allosteric, based on their mode of binding to a given receptor. An orthosteric ligand interacts with the same binding site as the natural endogenous agonist (neurotransmitter or hormone), while an allosteric ligand binds to another separate site (or sites) on the receptor. Allosteric ligands possess several advantages over orthosteric compounds. Allosteric binding sites on receptors have not faced the same evolutionary conservation pressure as orthosteric sites available to accommodate an endogenous neurotransmitter. Thus, allosteric sites show greater divergence among subtypes of a given receptor family, making it easier to develop selective allosteric modulators than orthosteric receptor antagonists or agonists. Moreover, pure allosteric modulators do not activate receptors on their own but just fine tune the action of the endogenous ligand, while preserving time and space patterns of physiological signalling. Therefore, allosteric modulators have been the subject of intensive research towards drug development, despite the challenges associated with detecting and quantifying often subtle allosteric effects[1,2].

Thanks to their relative simplicity and flexibility, radio-ligand binding techniques have become popular in studying drug-receptor interactions[3]. Fluorescence and bio-luminescence variants of ligand binding techniques were developed to overcome limitations of radio-ligand binding, like high non-specific binding of some radioligands[4]. However, radio-ligand binding studies are still widely used technique in functional characterization of G-protein coupled receptors[5], although they require more complex setup for studies of allosteric modulators. Ligand binding to a receptor is commonly defined by its dissociation constant at equilibrium (equilibrium dissociation constant). A single equilibrium dissociation constant exists for an orthosteric ligand, being defined as the ratio of the dissociation rate constant and association rate constant. In an allosteric system, ligands display two kinds of equilibrium dissociation constants: The “real” equilibrium dissociation constant for each ligand interacting with the unliganded receptor and the apparent equilibrium dissociation constant for a receptor in the presence of other ligands (orthosteric or allosteric). Another parameter that describes the behaviour of an allosteric system is the factor of binding cooperativity that denotes the maximal magnitude of change in the affinity of one ligand upon binding of the second ligand. In other words, the factor of cooperativity is a ratio between the apparent equilibrium dissociation constant for the receptor-ligand complex to that for the empty receptor. Thus, the apparent equilibrium dissociation constant is the product of the factor of cooperativity and equilibrium dissociation constant at the free receptor.

Many allosteric modulators display low affinity that renders them unsuitable to serve as radio-labelled ligands. Also, radio-labelled versions of allosteric modulators are often not commercially available or display enormous non-specific binding[6–9]. Under such circumstances, binding of allosteric modulators to the receptor is investigated indirectly using a radio-labelled orthosteric ligand as a tracer. Effects of increasing concentrations of an allosteric modulator on binding of a fixed concentration of a labelled orthosteric tracer allows determination of the equilibrium dissociation constant of an allosteric modulator and factor of cooperativity between an allosteric modulator and the tracer[10–13]. Several allosteric binding sites were discovered at various G-protein coupled receptors[14–18] as well as ionotropic receptors[19,20]. So, a researcher may face the question whether two allosteric modulators bind to the same site or to two distinct sites. In these cases, kinetic and hemi-equilibrium approaches are usually used[14,21,22]. Another possibility a researcher may encounter is bi-phasic tracer binding curves, including U-shaped and bell-shaped curves, suggesting the binding of one allosteric modulator at two allosteric sites on one receptor.

In this work, first, we derive equations describing equilibrium binding of an orthosteric tracer modulated by two allosteric modulators using their equilibrium dissociation constants and factors of cooperativity. Then we analyse the model under various scenarios and explore its limits. We show that under equilibrium conditions, binding of two allosteric modulators to the same site can be distinguished from binding to two separate sites. Further, we show that in the model of binding of two molecules of one allosteric modulator to one receptor only the apparent dissociation constants can be determined.

## Methods–Definition of models and derivation of equations

### Interaction of one orthosteric ligand and one allosteric ligand with a receptor

**Fig 1:**
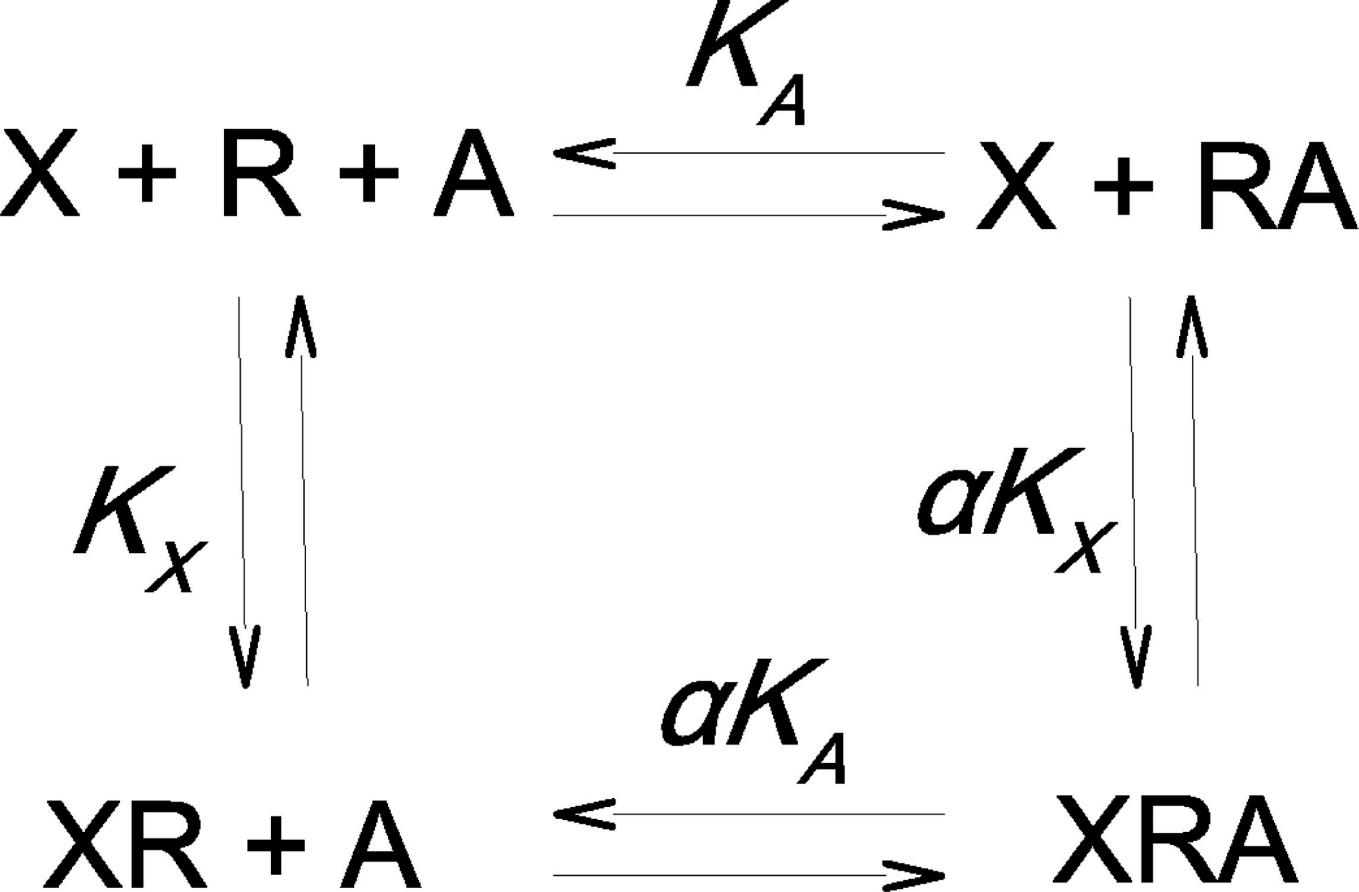
Ternary complex model. Allosteric interaction between tracer X and allosteric modulator A at receptor R. K_X_ is the equilibrium dissociation constant of tracer X in the absence of A, K_A_ is the equilibrium dissociation constant of allosteric modulator A in the absence of X and α is the factor of binding cooperativity between tracer and allosteric modulator.

Allosteric interaction is defined by concurrent binding of two ligands to the receptor to form a ternary complex (Fig 1). In this example, orthosteric ligand X (radiolabelled tracer) binds to the receptor R in the absence of allosteric modulator A with equilibrium dissociation constant K_X_, and the allosteric ligand A binds to R in the absence of the orthosteric ligand with an equilibrium dissociation constant K_A_. Subsequently, allosteric ligand A binds to the XR complex with equilibrium dissociation constant αK_A_ and the orthosteric tracer X binds to the RA complex with equilibrium dissociation constant αK_X_. Such interactions are depicted in Fig 1.

In case of positive cooperativity, equilibrium dissociation constants of the ternary complex XRA (αK_X_ and αK_A_) are smaller than the respective equilibrium dissociation constants (K_X_ and K_A_) of binary complexes XR and RA, respectively. Thus, the factor of cooperativity α is less than 1. In case of negative cooperativity, equilibrium dissociation constants of the ternary complex XRA (αK_X_ and αK_A_) are greater than respective equilibrium dissociation constants (K_X_ and K_A_) of binary complexes XR and RA, respectively. Thus, the factor of cooperativity α is greater than 1. In derivation of equations we adapted approach described by Ehlert[10] to calculate the ratio of tracer binding in the presence and in the absence of an allosteric modulator. Y, the fractional occupancy of the tracer X in the **absence** of the allosteric modulator A is described by Eq 1, where R_TOT_ is the total number of receptors:

$$Y=\frac{\left[X][R_{\mathrm{TOT}}\right]}{[X]{+K}_{X}}$$ Eq 1

Y’, the fractional occupancy of the tracer X in the **presence** of allosteric modulator A takes place according to Eq 2:

$$Y'=\frac{\left[X][R_{\mathrm{TOT}}\right]}{[X]+{K'}_{X}}$$ Eq 2

where K’X is the apparent equilibrium dissociation constant of tracer at a given concentration of A as described by Ehlert’s eq. A10 [10]:

$${K'}_{X}=K_{X}\frac{K_{A}+[A]}{K_{A}+\frac{\left[A\right]}{\alpha }}$$ Eq 3

that is an alternative expression of Hulme and Threvetick eq. 5a [12]. The ratio of binding of a fixed concentration of the tracer in the presence and in the absence of A follows this equation.

$$Y'/Y=\frac{[X]{+K}_{X}}{[X]{+K'}_{X}}$$ Eq 4

### Interaction of one orthosteric ligand and two allosteric ligands competing for a single allosteric site on a receptor

**Fig 2:**
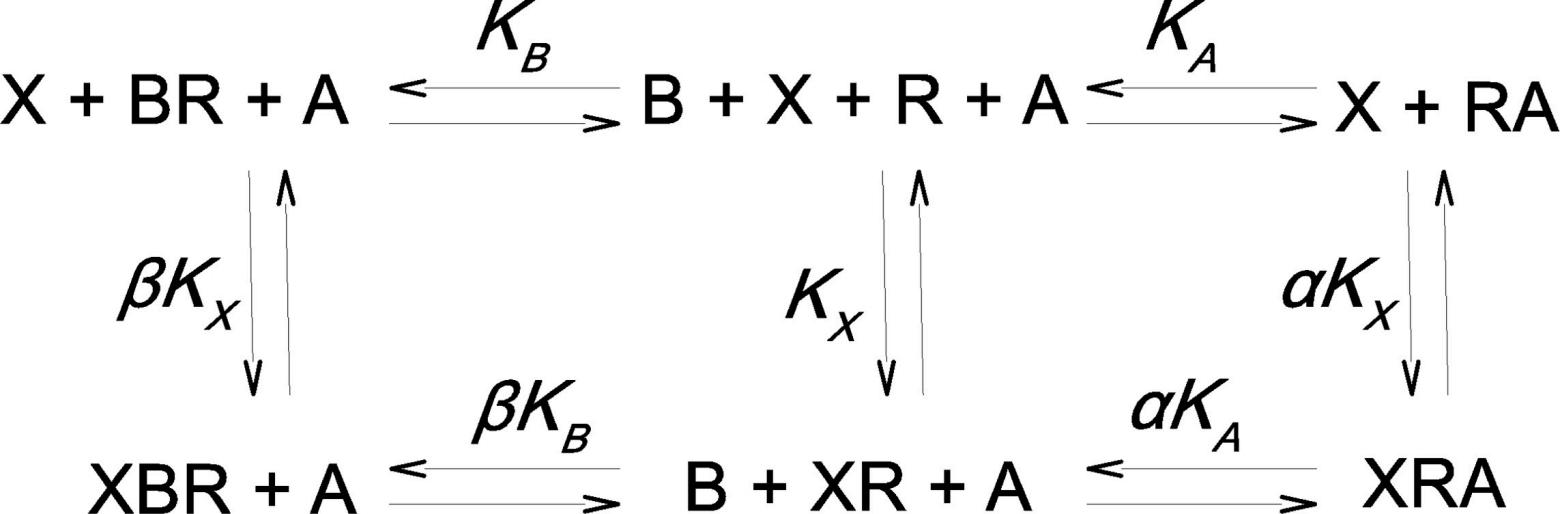
Two allosteric modulators binding to the same site. Allosteric interaction between tracer X and allosteric modulators A and B at receptor R, assuming that the two allosteric modulators compete for the same allosteric site. Beside parameters in *Fig 1*, K_B_ is the equilibrium dissociation constant of allosteric modulator B at the vacant receptor and β is the factor of binding cooperativity between tracer and allosteric modulator B.

Introduction of a second allosteric modulator B that competes for binding at the same site as the allosteric modulator A expands Fig 1 to Fig 2, where K_B_ is the equilibrium dissociation constant of B for R and β is factor of cooperativity between tracer binding and binding of allosteric modulator B. The apparent equilibrium dissociation constant of tracer, K’_X_, in the presence of a given concentration of both A and B is given by Eq 5 (S1 Text [A22]):

$${K'}_{X}=K_{X}\frac{1+\frac{\left[A\right]}{K_{A}}+\frac{\left[B\right]}{K_{B}}}{1+\frac{\left[A\right]}{{\alpha K}_{A}}+\frac{\left[B\right]}{{\beta K}_{B}}}$$ Eq 5

### Interaction of an orthosteric tracer and two allosteric ligands each binding to its own site on a receptor

**Fig 3:**
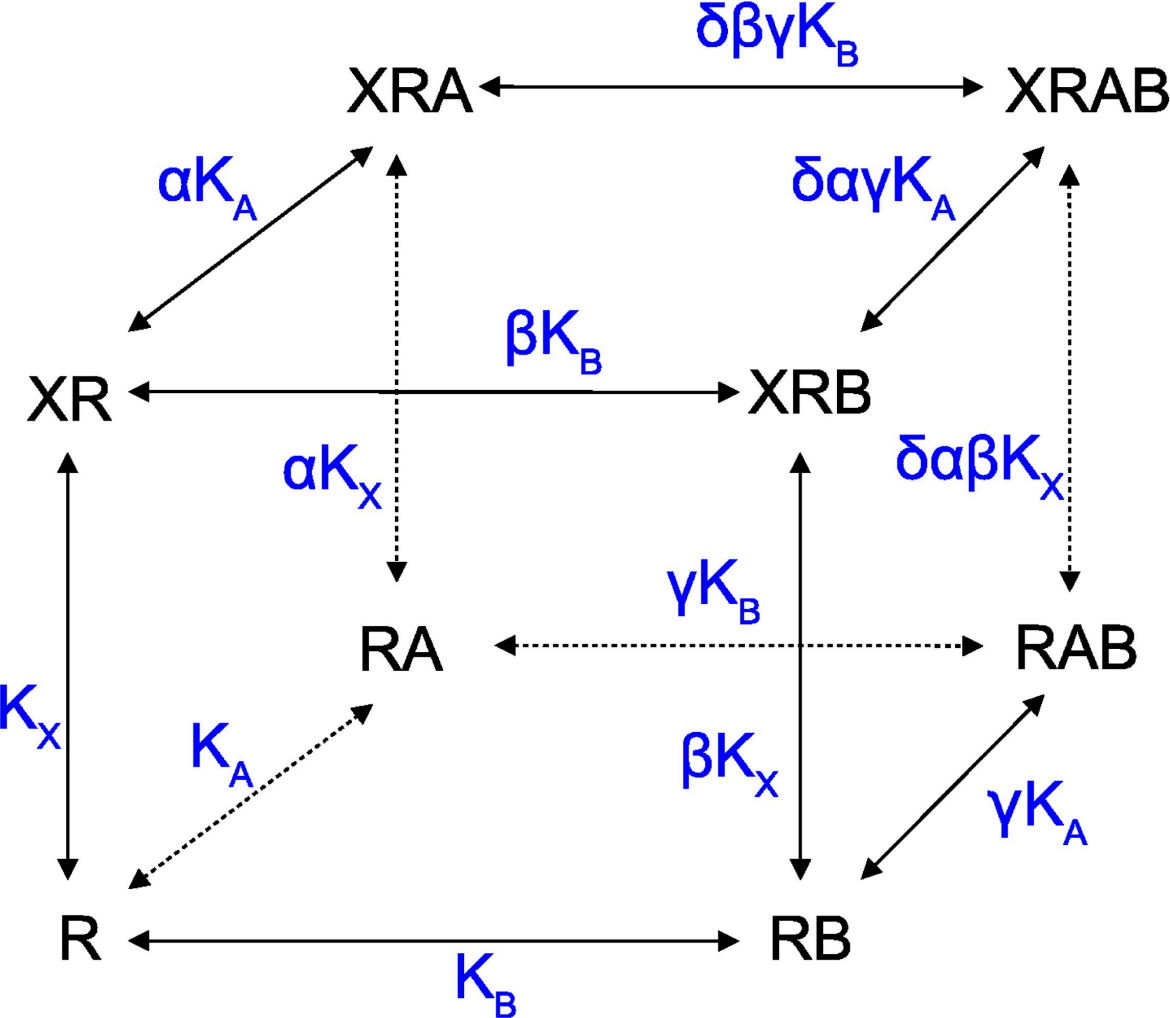
Two allosteric modulators each binding to its own site. Allosteric interaction between tracer X and allosteric modulators A and B at receptor R. Each allosteric modulator binds to its own site, with cooperative interaction between the two sites. Besides parameters listed in Figs *1* and *2*, γ is the factor of binding cooperativity between modulator A and modulator B and δ is the change in factor cooperativity γ caused by tracer binding.

In case each of two allosteric modulators binds to its own site on the receptor, and the two sites interact with each other allosterically, Fig 2 expands to Fig 3, where five additional reactions are possible and two additional parameters need to be introduced. The factor of cooperativity γ quantifies maximal bidirectional modulation of binding of the two allosteric agents by each other. The cooperativity factor δ quantifies the change to the pairwise factors of cooperativity (α, β and γ) when all three ligands are bound to the receptor. Due to the law of microscopic reversibility, the final cooperativity must be the same regardless of the sequence of steps taken to form the quaternary complex XRAB. Let λ be a change in the factor of cooperativity γ upon binding of tracer X to the RAB complex, κ is a change in the factor cooperativity β upon binding of the modulator A, and ζ is a change in the factor of cooperativity α upon binding of modulator B. Then, λ*γ, κ*β, and ζ*α and δ must be equal (See S1 Text). The apparent equilibrium dissociation constant of tracer K’_X_ in the presence of the two allosteric modulators A and B is then given by Eq 6 (S1 Text [A44]):

$${K'}_{X}=K_{X}\frac{1+\frac{\left[A\right]}{K_{A}}+\frac{\left[B\right]}{K_{B}}(1+\frac{\left[A\right]}{{\gamma K}_{A}})}{1+\frac{\left[A\right]}{{\alpha K}_{A}}+\frac{\left[B\right]}{{\beta K}_{B}}(1+\frac{\left[A\right]}{{\alpha \gamma \delta K}_{A}})}$$ Eq 6

### Interaction of the orthosteric tracer and one allosteric ligand that binds to two allosteric sites with different affinities

Bi-phasic tracer binding curves in the presence of increasing concentrations of an allosteric modulator indicate binding of two molecules of the allosteric modulator to one receptor. To this end Eq 6 can be reformulated to give Eq 7:

$$K_{X}\frac{1+\frac{\left[A\right]}{K_{A1}}+\frac{\left[A\right]}{K_{A2}}(1+\frac{\left[A\right]}{{\gamma K}_{A1}})}{1+\frac{\left[A\right]}{\alpha_{1}K_{A1}}+\frac{\left[A\right]}{\alpha_{2}K_{A2}}(1+\frac{\left[A\right]}{\alpha_{1}{\gamma \delta K}_{A1}})}$$ Eq 7

where K_A1_ and K_A2_ are equilibrium dissociation constants of the allosteric modulator for respective sites and α_1_ and α_2_ are the corresponding factors of cooperativity with binding of the tracer.

## Results–Analysis of models

### Interaction of one orthosteric ligand and two allosteric ligands competing for a single allosteric site on a receptor (Fig 2)

**Fig 4:**
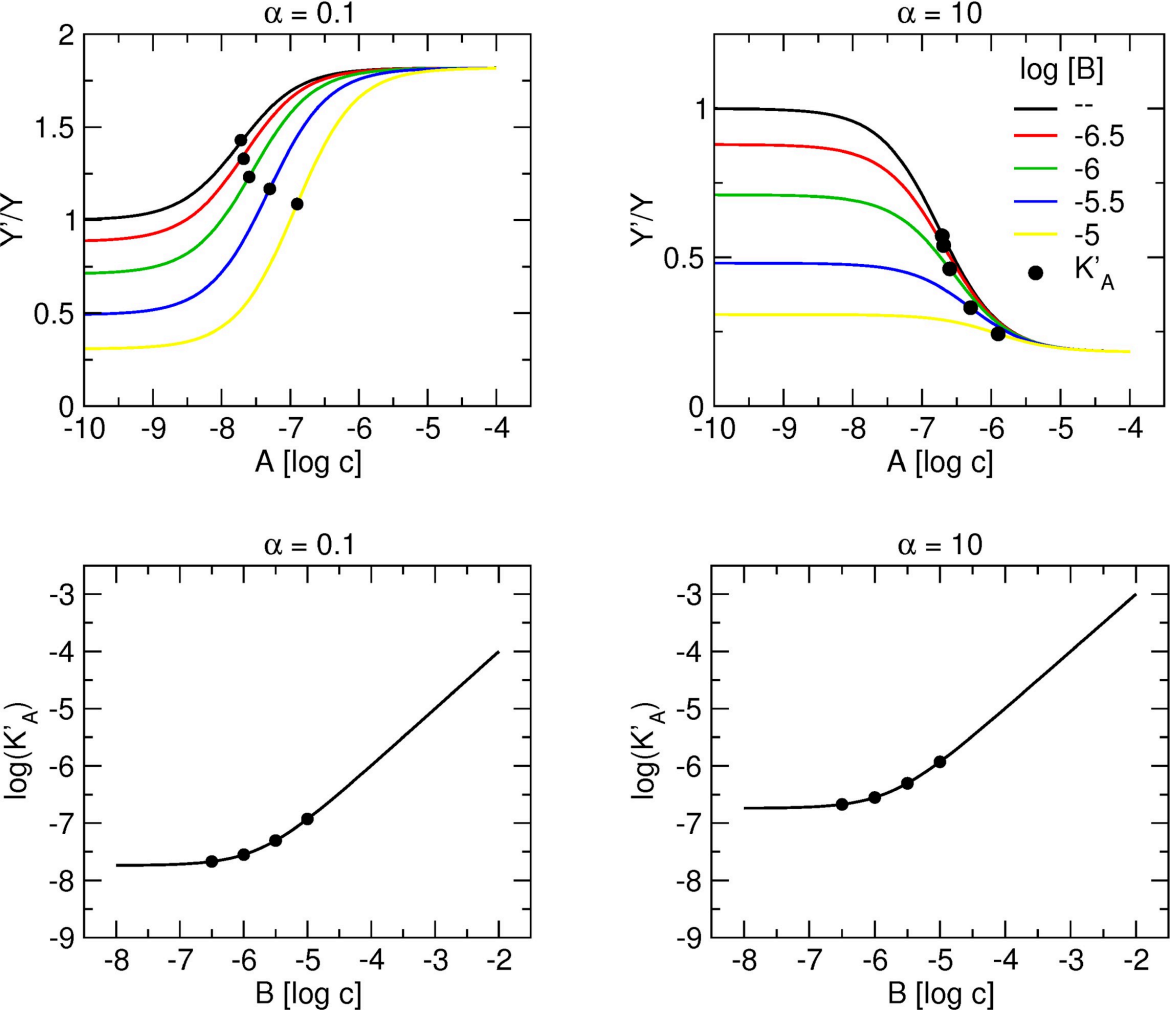
Concentration dependence of the interaction of two allosteric modulators that compete for the same allosteric site. Upper row, simulation of tracer binding (ordinate) in the presence of two allosteric modulators A and B competing for the same allosteric site, where α is either less than one (left) or greater than one (right) and B is negative allosteric modulator. Binding of the tracer is expressed as the ratio to the binding in the absence of allosteric modulators. Abscissa, concentration of allosteric modulator A expressed as logarithm of molar concentration. Logarithm of concentration of allosteric modulator B is shown in the graph legend. Simulation parameters: K_X_ = 0.1 nM, [X] = 0.1 nM, K_A_ = 100 nM, K_B_ = 1 μM, β = 10. Lower row, dependence of the apparent equilibrium dissociation constant of modulator A (K’_A_) on concentration of modulator B.

Fig 4 illustrates binding of a tracer at a concentration equal to its K_X_ in the presence of increasing concentrations of the allosteric modulator A with positive (left) or negative (right) binding cooperativity α in the absence and in the presence of a fixed concentration of the allosteric modulator B with negative binding cooperativity β. Importantly, all curves end at the same level because when the concentration of A exceeds that of B more than 100-times, Eq 5 approximates Eq 8:

$${K'}_{X}=K_{X}\frac{1+\frac{\left[A\right]}{K_{A}}}{1+\frac{\left[A\right]}{{\alpha K}_{A}}}$$ Eq 8

that after rearrangement results in Eq 3. In other words, at high concentrations, allosteric modulator A displaces all binding of modulator B and tracer binding is the same as in the absence of B.

By rearrangement of equation Eq 5, the apparent equilibrium dissociation constant of A (K’_A_) can be calculated according to Eq 9:

$${K'}_{A}=K_{A}\frac{1+\frac{\left[X\right]}{K_{X}}+\frac{\left[B\right]}{K_{B}}(1+\frac{\left(X\right]}{{\beta K}_{X}})}{1+\frac{\left[X\right]}{{\alpha K}_{X}}}$$ Eq 9

Concentration dependency of K’_A_ on the concentration of B is illustrated in the lower row of Fig 4 for a concentration of the tracer equal to its K_X_. At saturating concentrations of B, the relation between K’_A_ and concentration of B is linear with slope equal to one.

### Interaction of an orthosteric tracer and two allosteric ligands each binding to its own site on a receptor (Fig 3)

**Fig 5:**
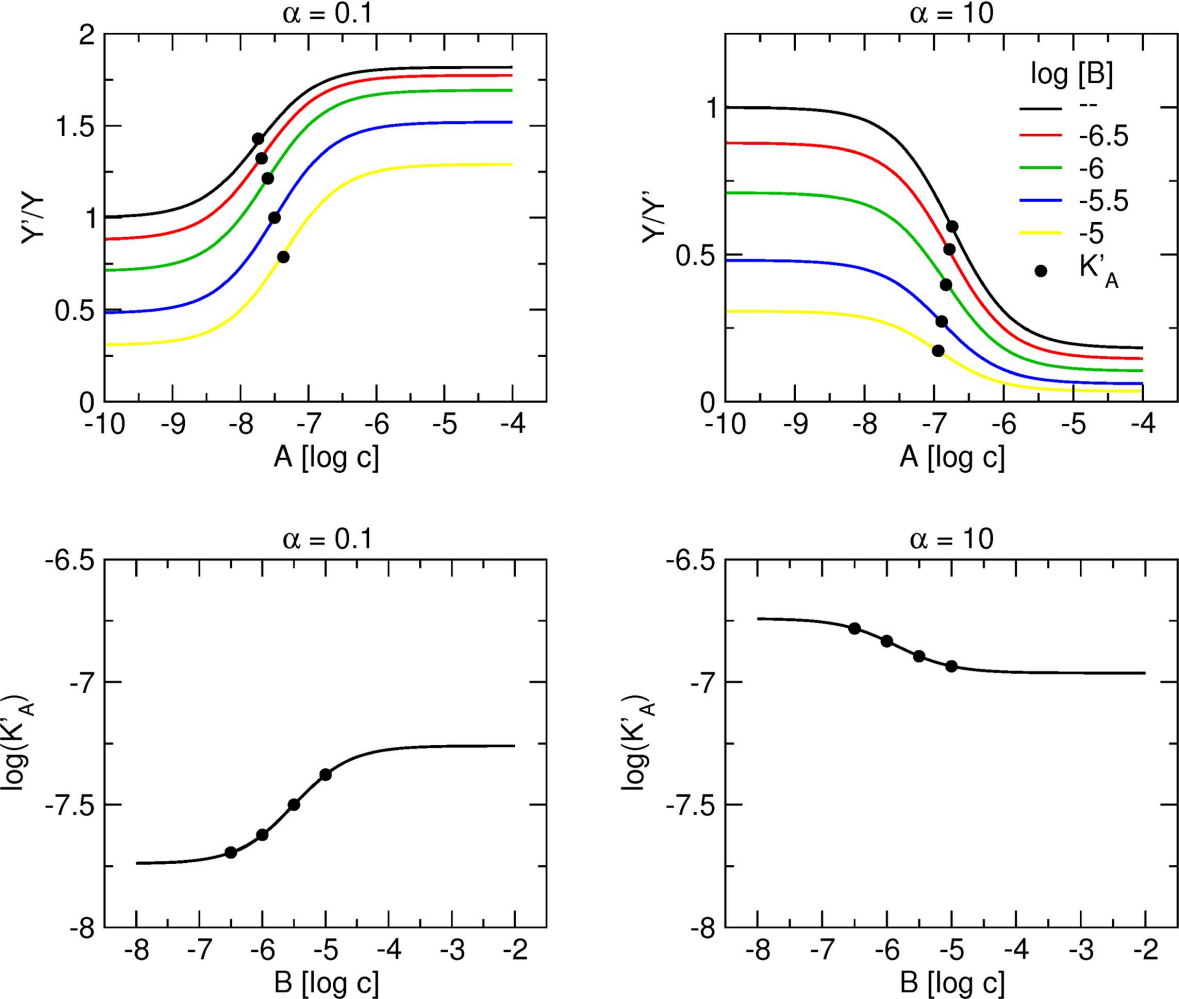
Concentration dependence of the interaction of two allosteric modulators binding each to its own allosteric site. Upper row, simulation of tracer binding (ordinate) in the presence two allosteric modulators A and B each binding to its own site, where A is either positive (left) or negative (right) and B is negative allosteric modulator. Binding of the tracer is expressed as the ratio to the binding in the absence of allosteric modulators. Abscissa, concentration of allosteric modulator A expressed as logarithm of molar concentration. Logarithm of concentration of allosteric modulator B is shown in the graph legend. Simulation parameters: K_X_ = 0.1 nM, [X] = 0.1 nM, K_A_ = 100 nM, K_B_ = 1 μM, β = 10, γ = 1, δ = 1. Lower row, dependence of the apparent equilibrium dissociation constant of modulator A (K’_A_) on concentration of modulator B.

The upper row of Fig 5 illustrates binding of tracer at a concentration equal to its K_X_ in the presence of increasing concentrations of allosteric modulator A with positive (left) or negative (right) binding cooperativity α in the absence and in the presence of allosteric modulator B with negative binding cooperativity β. Modulators A and B bind to their unique respective sites on the receptor and interact with each other allosterically. In this example the factor of binding cooperativity γ (between modulators A and B) is equal to 1 (neutral) and thus binding of modulator A does not affect binding of modulator B and vice versa. Unlike in the case of competition of A and B for the same site, tracer binding curve in the absence of modulator B is parallel to the one in its presence.

By rearrangement of equation Eq 6, the apparent equilibrium dissociation constant of A (K’_A_) can be calculated according to Eq 10:

$${K'}_{A}=K_{A}\frac{1+\frac{\left[B\right]}{K_{B}}+\frac{\left[X\right]}{K_{X}}(1+\frac{\left[B\right]}{{\beta K}_{B}})}{1+\frac{\left[B\right]}{{\gamma K}_{B}}+\frac{\left[X\right]}{{\alpha K}_{X}}(1+\frac{\left[B\right]}{{\beta \gamma \delta K}_{B}})}$$ Eq 10

Concentration dependency of K’_A_ on the concentration of B is illustrated in the lower row of Fig 5 for a concentration of the tracer equal to its K_X_. At saturating concentrations of B, K’_A_ approaches the limit given by Eq 10 that is equal to K_A_ for zero concentration of X. In case of α < 1, K’_A_ rises from α*K_A_ value in the absence of B towards K_A_ value (Fig 5, lower left). In case of α > 1, K’_A_ declines from α*K_A_ value in the absence of B towards K_A_ value (Fig 5, lower right).

**Fig 6:**
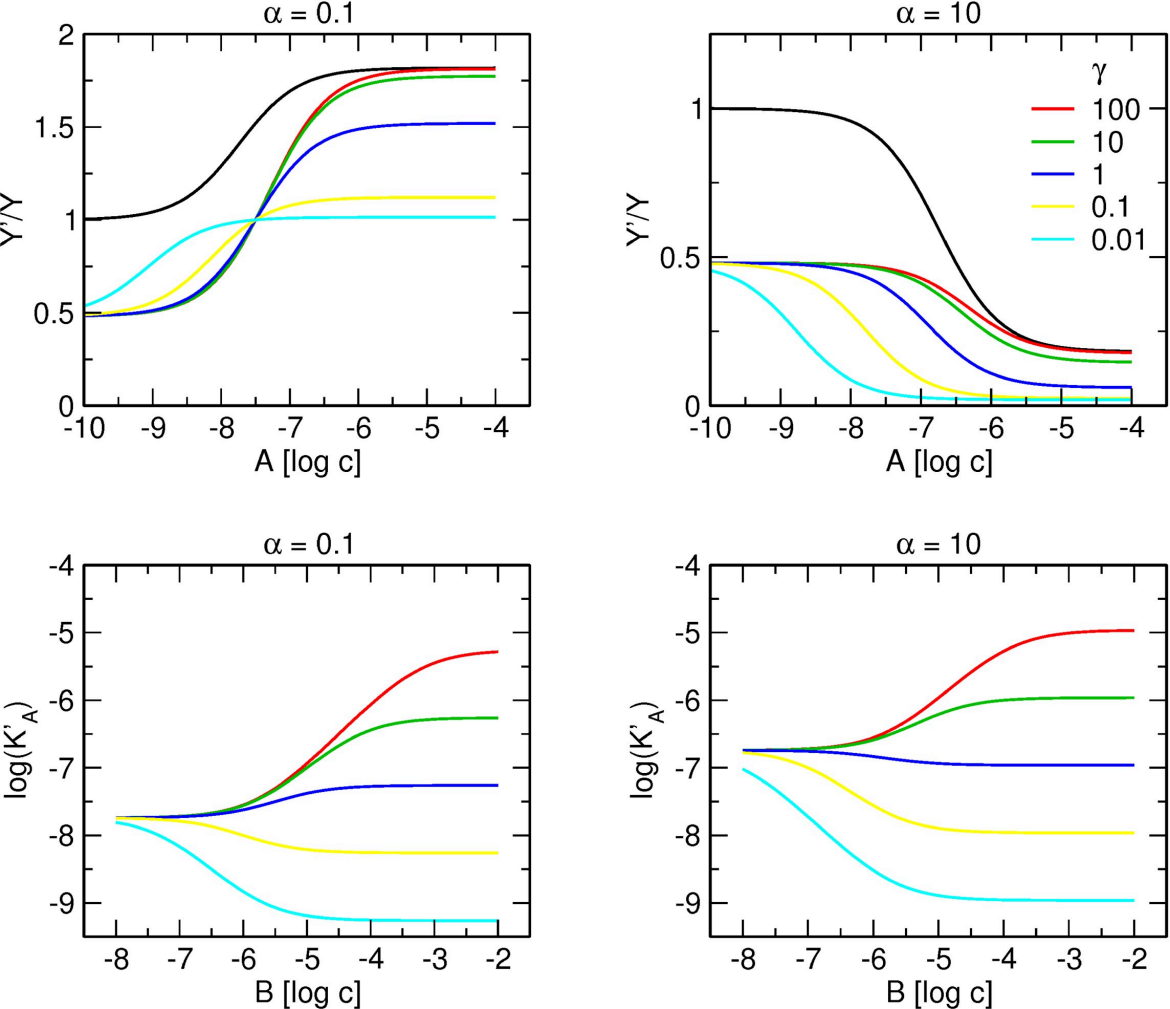
Effect of cooperativity factor γ on the interaction of two allosteric modulators binding each to its own allosteric site. Upper row, simulation of tracer binding (ordinate) in the presence two allosteric modulators A and B each binding to its own site, where A is either positive (left) or negative (right) and B is negative allosteric modulator. Binding of the tracer is expressed as the ratio to the binding in the absence of allosteric modulators. Abscissa, concentration of allosteric modulator A expressed as logarithm of molar concentration. Value of cooperativity factor γ is shown in the graph legend. Simulation parameters: K_X_ = 0.1 nM, [X] = 0.1 nM, K_A_ = 100 nM, K_B_ = 1 μM, β = 10, log[B] = -5.5, δ = 1. Lower row, dependence of the apparent equilibrium dissociation constant of modulator A (K’_A_) on concentration of modulator B for various values of cooperativity factor γ.

In case of negative cooperativity γ (between modulators A and B) the curve in the presence of modulator B approaches control curve (in the absence of modulator B) (Fig 6). Due to negative cooperativity (γ >1), the effect of modulator B becomes smaller with an increase in the concentration of modulator A. In this case, a decrease in tracer binding caused by modulator B is smaller and the two curves approach each other. In Eq 6, for extremely high values of γ the expression (1 + [A]/(γK_A_)) and expression (1 + [A]/(αγδK_A_)) are virtually 1 and Eq 6 transforms into Eq 5. In practice, a difference in binding smaller than 5% may be hard to detect and thus it may be difficult to distinguish competition between modulators A and B from allosteric interaction with the factor of cooperativity γ greater than 10. In such case, measurements at high concentrations of modulator B may be needed to make the incomplete approach of curves visible. Alternatively, analysis of the apparent affinity of modulator A (K’_A_) at various high concentrations of B may be necessary (Fig 6, lower row). Non-linearity at high concentrations of B may indicate saturation of its effect on K’_A_ and thus the allosteric nature of interaction between A and B. In case of positive cooperativity between allosteric modulators A and B, the curve in the presence of modulator B departs from the control curve (Fig 6, upper row) and K’_A_ declines with an increase in the concentration of B (Fig 6, lower row).

**Fig 7:**
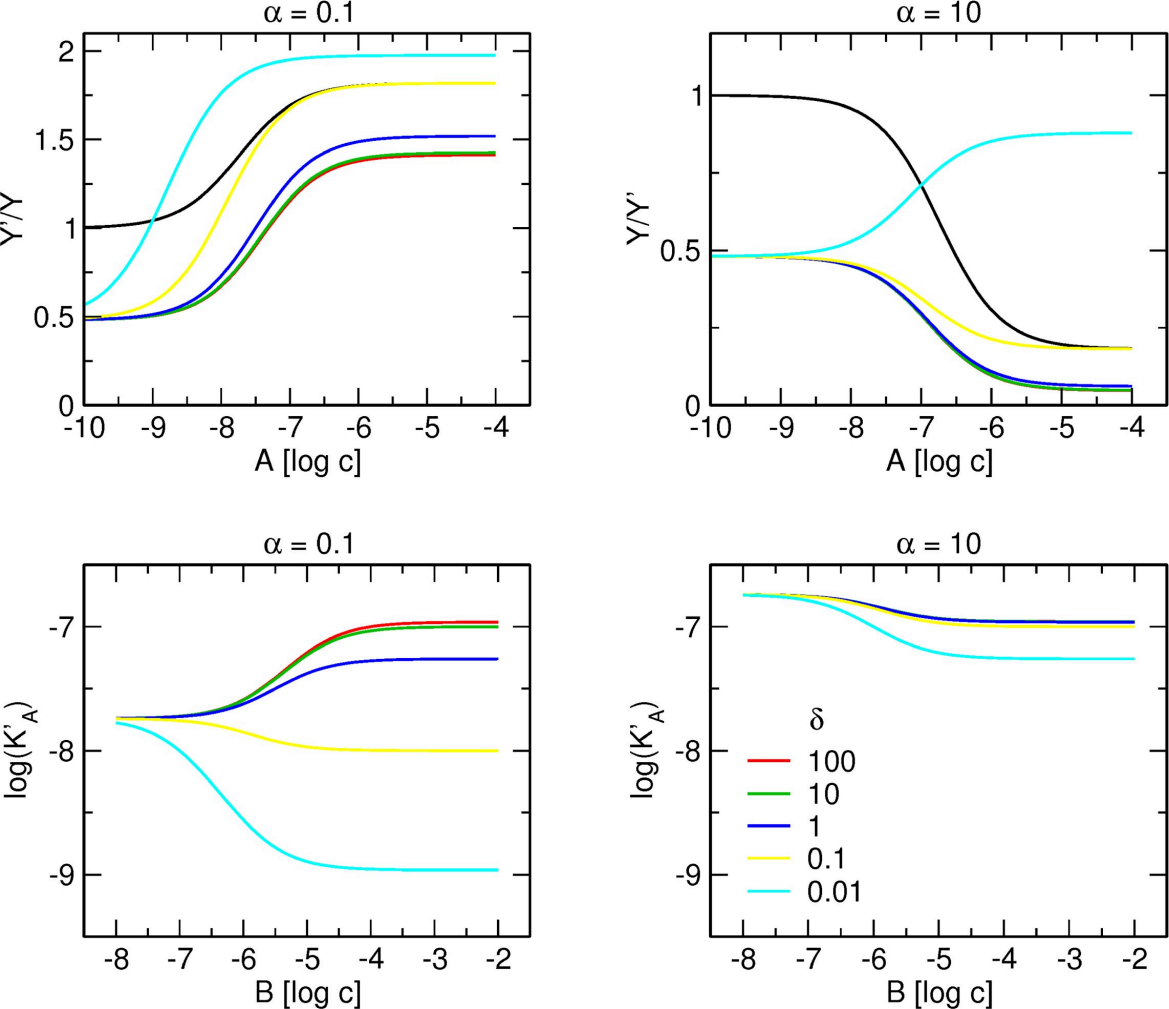
Effect of cooperativity factor δ on the interaction of two allosteric modulators binding each to its own allosteric site. Upper row, simulation of tracer binding (ordinate) in the presence two allosteric modulators A and B each binding to its own site, where A is either positive (left) or negative (right) and B is negative allosteric modulator. Binding of the tracer is expressed as the ratio to the binding in the absence of allosteric modulators. Abscissa, concentration of allosteric modulator A expressed as logarithm of molar concentration. Value of cooperativity factor δ is shown in the graph legend. Simulation parameters: K_X_ = 0.1 nM, [X] = 0.1 nM, K_A_ = 100 nM, K_B_ = 1 μM, β = 10, log[B] = -5.5, γ = 1. Lower row, dependence of the apparent equilibrium dissociation constant of modulator A (K’_A_) on concentration of modulator B for various values of cooperativity factor δ.

Effects of cooperativity factor δ are illustrated in Fig 7. Effects of δ values smaller than 1 on tracer binding are much more evident than effects of δ values greater than 1. Values of δ < 1 lead to an increase in tracer binding that is dependent on the concentration of modulator A (Fig 7, upper row) and to a decrease in the apparent equilibrium dissociation constant of modulator A (K’_A_) that is dependent on the concentration of modulator B (Fig 7, lower row). A certain δ value may result in convergence of curves in the presence of modulator B (yellow) with the control curve (black) in the absence of modulator B. Provided that γ is equal to 1, convergence of the binding curves in the absence and in the presence of allosteric modulator B occurs when δ is equal to the reciprocal value of β. At even lower δ values, binding in the presence of modulator B (cyan) surpasses control binding in the absence of modulator B (black). Effects of δ value on K’_A_ are evident only when it is greater than the combined effects of α, β and γ (Fig 7, lower row). When the combined effects of α, β and γ are neutral (0.1 * 10 * 1 = 1; Fig 7, lower left), values of δ > 1 increase K’_A_ and values of δ < 1 decrease K’_A_. On the other hand when the combined effects of α, β and γ is markedly negative (10 * 10 * 1 = 100; Fig 7, lower right), only very low values of δ decrease K’_A_.

**Fig 8:**
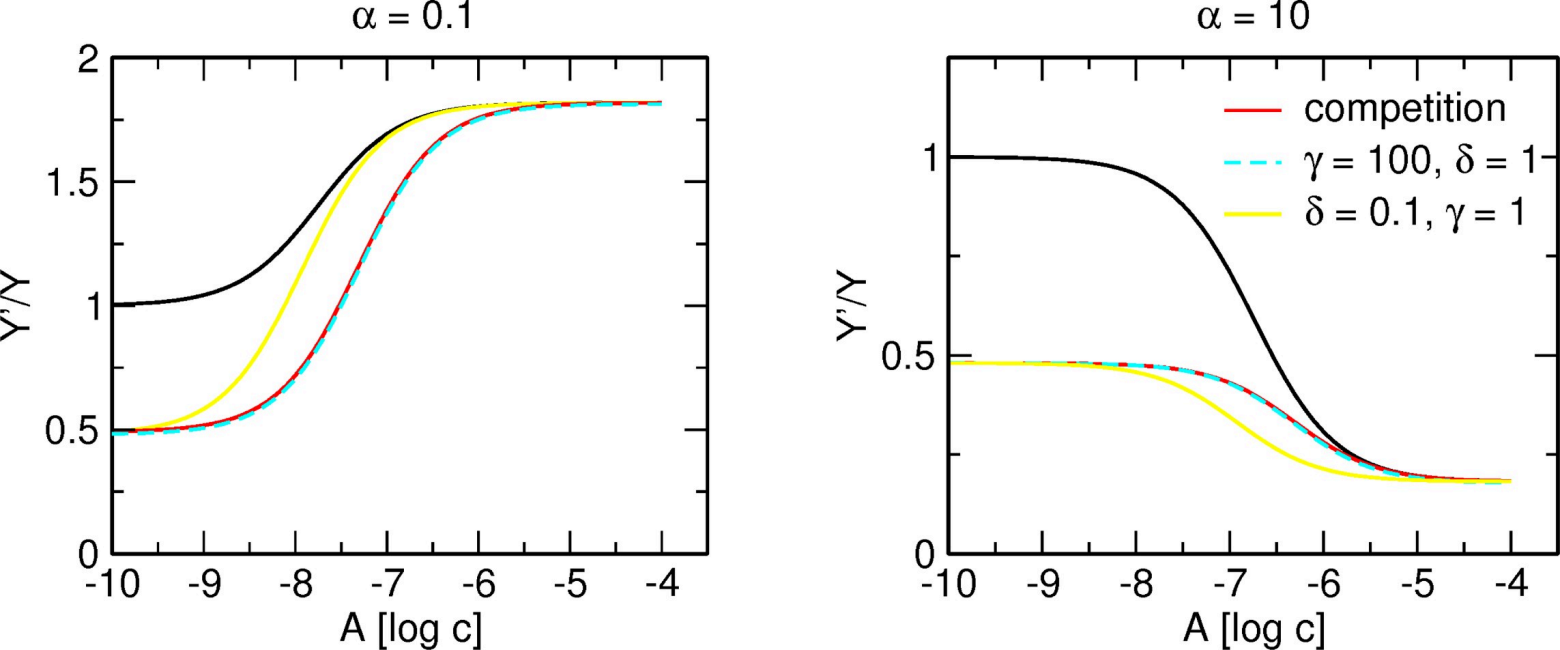
**Comparison of competition and allosteric interaction between modulators A and B.** Simulation of tracer binding (ordinate) in the presence two allosteric modulators A and B each binding to its own allosteric site (yellow line) or competing for the same allosteric site (red line), where A is either positive (left) or negative (right) and B is negative allosteric modulator. Black curve, tracer binding in the absence of modulator B. Binding of the tracer is expressed as fold over control binding in the absence of allosteric modulators. Abscissa, concentration of allosteric modulator A expressed as logarithm of molar concentration. Simulation parameters: K_X_ = 1 nM, [X] = 1 nM, K_A_ = 100 nM, K_B_ = 1 μM, β = 10, log[B] = -5.5.

In practice, K_X_ value should be determined first in saturation experiments, then in separate experiments parameters of modulator A (K_A_ and α) and modulator B (K_B_ and β) would be determined using Eq 3. A simple way to judge whether two allosteric modulators compete for the same site is to compare theoretical curves calculated according to Eq 5 to experimental data (e.g., using a run test) for the goodness of fit. However, as mentioned above, tracer binding curves in the presence and in the absence of allosteric modulator B may converge even when modulators A and B do not compete for the same site. If a convergence is due to low δ value (Fig 8, yellow), the curve is located to the left from the curve corresponding to competition between modulators A and B (red). This is because apparent K’_A_ is the product of δ and K_A_, according to Eq 8. In this case, a test of goodness of fit to Eq 5 would fail, pointing to identification of allosteric interaction between A and B. However, strong negative cooperativity between allosteric modulators A and B (cyan) is indistinguishable from competition. A high value of γ makes the multiplier (enclosed in parenthesis) in the denominator of Eq 6 equal to 1 and thus Eq 6 becomes analogous to Eq 5. In this case, a test of goodness of fit to Eq 5 would pass and falsely indicate competition between A and B. To avoid false indications of competition between A and B, the apparent equilibrium dissociation constant of modulator A at various high concentrations of modulator B must be determined. In case of competition between modulators A and B, dependence of K’_A_ values on the concentration of B follows Eq 9. However, in case of allosteric interaction between modulators A and B, dependence of K’_A_ values on the concentration of B follows Eq 10.

To determine values of γ and δ with all remaining parameters being fixed, Eq 6 should be fitted to experimental data. It should be noted that low concentrations of modulator B lead to underestimation of γ value and high concentrations of modulator B lead to overestimation of δ value. At low concentrations of modulator B, the factor of cooperativity γ is underestimated because the effect of modulator B is weak and therefore a difference between the apparent and real equilibrium dissociation constants may be obscured by signal noise. At high concentrations of modulator B, the factor of cooperativity δ is overestimated because a small error in the determination of the second plateau translates into a large change in δ value. Thus, a global fit of Eq 6 (3-D fit with B as the second variable) to several curves measured at various concentrations of modulator B may be needed to reliably determine values of γ and δ. It should be noted that the cooperativity factors γ and δ may have similar effects on tracer binding. To tell the effects of γ and δ factors apart measurement of tracer binding at various combinations of concentrations of tracer and allosteric modulator B followed by a global fit of Eq 6 (4-D fit with X and B as the second and the third variable) may be needed.

Alternatively, analysis of K’_A_ values may be performed to decrease the number of degrees of freedom and to increase the robustness of the fitting procedure. To this aim, the values of K’_A_ for various combinations of concentrations of tracer X and allosteric modulator B need to be obtained by fitting a logistic equation with variable slope to individual data sets. Then, a global fit of Eq 10 to calculated K’_A_ values (4-D fit with X and B as the second and the third variable) has to be performed.

### Interaction of the orthosteric tracer and one allosteric ligand that binds to two allosteric sites with different affinities

**Fig 9:**
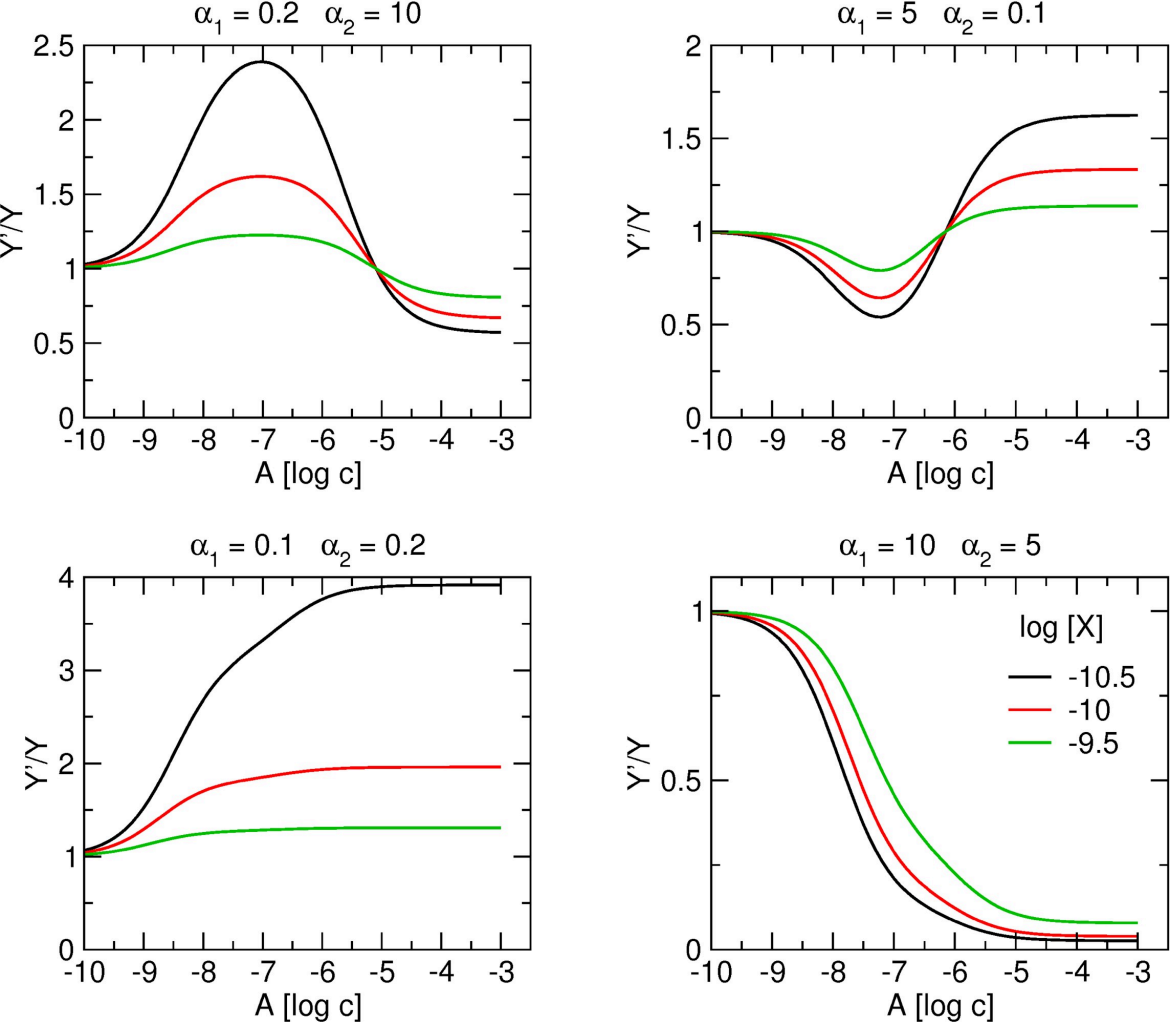
Concentration dependence of binding of allosteric modulator to two allosteric sites. Simulation of tracer binding (ordinate) in the presence an allosteric modulator binding to two allosteric sites for various combinations of positive and negative cooperativity with the tracer indicated at headings of individual plots. Binding of the tracer is expressed as fold over control binding in the absence of allosteric modulator. Abscissa, concentration of allosteric modulator expressed as logarithm of molar concentration. Logarithm of concentration of tracer X is shown in the graph legend. Simulation parameters: K_X_ = 0.1 nM, K_A1_ = 10 nM, K_A2_ = 1 μM, γ = 1, δ = 1.

Tracer binding modulated by two molecules of the same allosteric modulator is illustrated in Figs 6, 7 and 8. Fig 9 shows the effects of tracer concentration on the shape of the tracer binding curve. Allosteric effects are more pronounced at low concentrations of the tracer. At any concentration of the tracer, the apparent equilibrium dissociation constant for high- and low-affinity binding sites remains constant, curves intersect at inflexion points. A biphasic nature of binding curves is well observed when the cooperativity with tracer from individual allosteric sites has opposite directions (being positive at one and negative at the other; Fig 6, upper graphs) as compared to being in the same direction (either positive or negative; Fig 6, lower graphs). In the latter case, strong cooperativity from the site with higher affinity may obscure weak cooperativity from the site with lower affinity.

**Fig 10:**
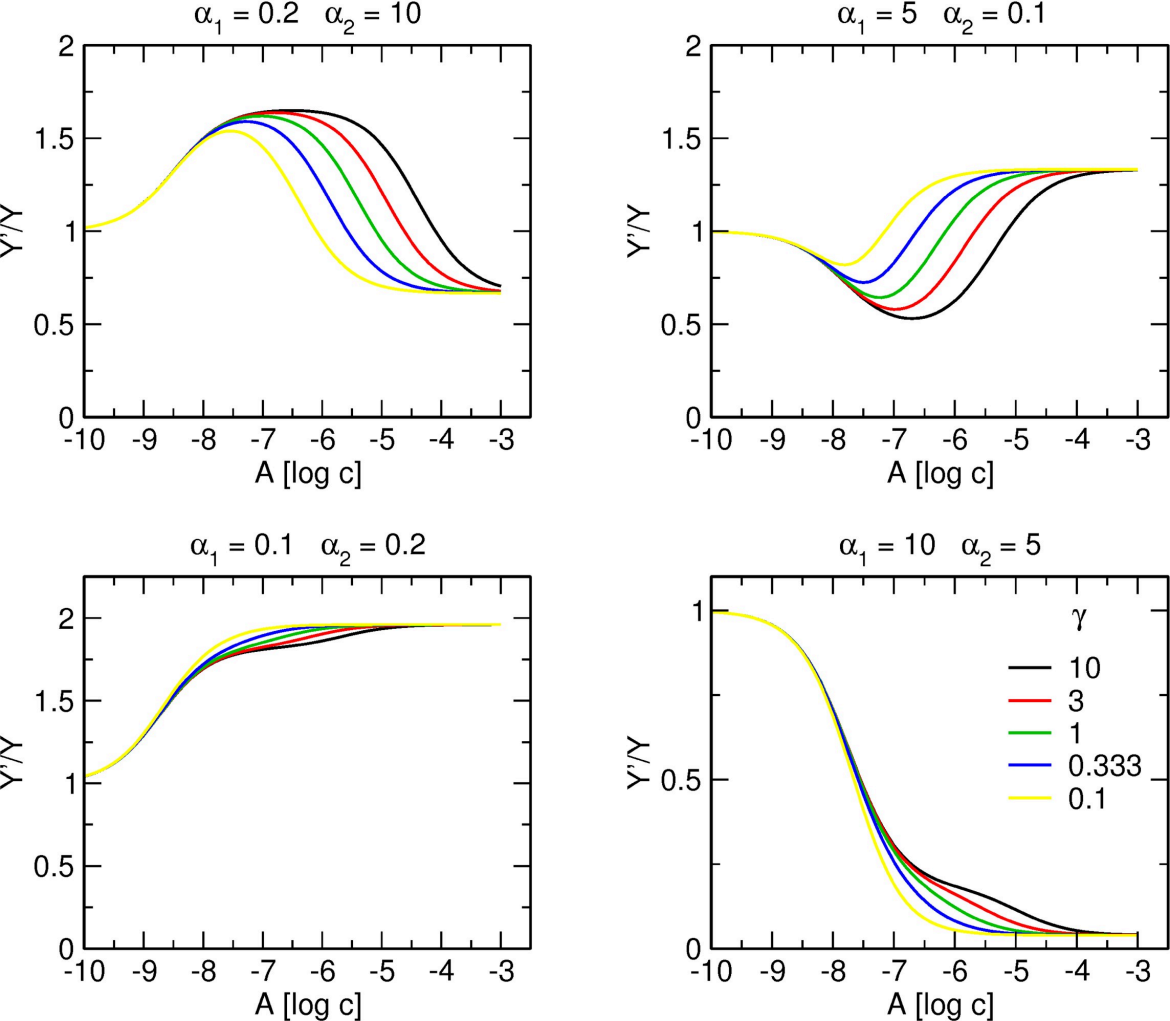
Effect of γ value on binding of allosteric modulator to two allosteric sites. Simulation of tracer binding (ordinate) in the presence an allosteric modulator binding to two allosteric sites for various combinations of positive and negative cooperativity with the tracer indicated at headings of individual plots. Binding of the tracer is expressed as fold over control binding in the absence of allosteric modulator. Abscissa, concentration of allosteric modulator expressed as logarithm of molar concentration. Value of cooperativity factor γ is shown in the graph legend. Simulation parameters: K_X_ = 0.1 nM, [X] = 0.1 nM K_A1_ = 100 nM, K_A2_ = 1 μM, δ = 1.

Fig 10 shows the effects of the factor of cooperativity γ between binding of individual molecules of an allosteric modulator on tracer binding. The factor of cooperativity γ influences the apparent equilibrium dissociation constant of the low-affinity binding site K’_A2_. It brings the two phases of binding curves closer when γ < 1 and puts them further apart when γ > 1. In case these two phases are so close that no plateau between them is established then maximal change in tracer binding elicited from the high-affinity binding site is affected by binding of an allosteric modulator to the low-affinity site. Consequently, the effect of allosteric modulator at the high-affinity site is not fully developed and a change in tracer binding (either increase or decrease) is smaller. The α_1_ value is determined from the maximal change elicited from binding of the allosteric agent to the high-affinity binding site. When a plateau is not established, the value of α_1_ cannot be reliably estimated. Consequently, K_A1_ cannot be reliably estimated either as apparent K’_A1_ (first inflexion point) is a product of α_1_ * γ * δ * K_A1_. On the other hand, a plateau between phases is a sign that binding of an allosteric modulator to the high-affinity site is not affected by binding of the second molecule to the low-affinity site. In such case, γ and δ can be neglected and K_A1_ and α_1_ can be reliably determined by fitting Eq 3 to the first phase of a binding curve, including a plateau.

In case the two allosteric sites have a similar affinity, γ values may obscure the biphasic nature of the binding curve. When the direction of cooperativity with tracer from individual allosteric sites is in the same direction (Fig 10, lower graphs) and the two allosteric sites have a similar affinity, very low values of γ are manifested by steep binding curves with slope factor greater than 1.

**Fig 11:**
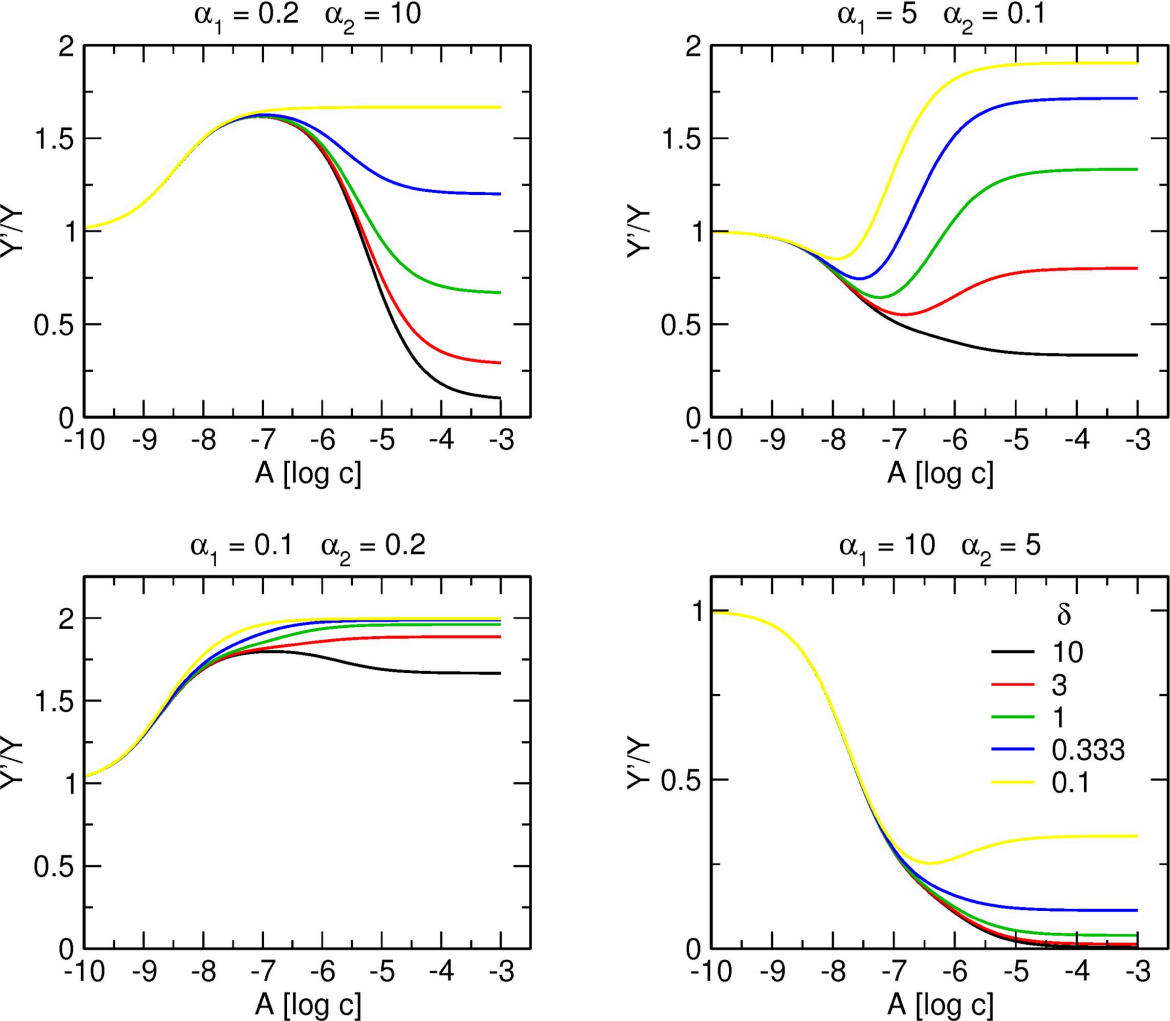
Effect of δ value on binding of allosteric modulator to two allosteric sites. Simulation of tracer binding (ordinate) in the presence an allosteric modulator binding to two allosteric sites for various combinations of positive and negative cooperativity with the tracer indicated at headings of individual plots. Binding of the tracer is expressed as fold over control binding in the absence of allosteric modulator. Abscissa, concentration of allosteric modulator expressed as logarithm of molar concentration. Value of cooperativity factor δ is shown in the graph legend. Simulation parameters: K_X_ = 0.1 nM, [X] = 0.1 nM K_A1_ = 10 nM, K_A2_ = 1 μM, γ = 1.

Fig 11 shows the effects of δ value on the shape of a tracer binding curve. Cooperativity factor δ is a change in α_2_ by allosteric modulator binding to the high-affinity site. Thus, the value of δ determines the level of the plateau of the second phase of the tracer binding curve. Apparent α’_2_ value is a product of δ * α_2_ and apparent K’_A2_ value is a product of α_2_ * γ * δ * K_A2_. Unlike K_A1_ and α_1_ that can be determined in case of separation of the two binding phases by a plateau, apparent values of K’_A2_ and α’_2_ of the low-affinity site are always affected by binding of an allosteric modulator to the high-affinity site and thus cannot be broken down to their principal components α_2_, γ, δ, and K_A2_.

Parameter δ has quite a profound effect on the shape of the binding curve. High δ values may enhance negative cooperativity of the low-affinity site (Fig 11, upper left, green vs. black) or turn positive cooperativity of the low-affinity site to negative cooperativity (Fig 11, upper right, green vs. black). Low δ values may enhance positive cooperativity of low-affinity site (Fig 11, lower left, green vs. yellow) or turn negative cooperativity at the low-affinity site to positive cooperativity (Fig 11, lower right, green vs. yellow).

## Discussion

In the present study, we provide mathematical analysis of equilibrium binding of an orthosteric tracer modulated by two allosteric ligands. Allosteric modulators possess several advantages over orthosteric agonists and antagonists, especially in regard to higher selectivity for one receptor subtype versus another. Therefore, allosteric modulators are the subject of intensive research in drug development[1,2]. Proper determination of affinity and other binding parameters of allosteric modulators is essential for the process of drug discovery.

Equilibrium binding studies using a fixed concentration of a tracer can be used to measure binding parameters of allosteric modulators, such as their affinities, and magnitude and direction of their cooperativity with tracer[10]. However, it should be verified in kinetic experiments that the system is indeed at equilibrium[12]. The existence of multiple allosteric binding sites at several G-protein coupled receptors has been postulated[14–18]. Analysis of the effects of a combination of two allosteric modulators in radioligand binding assays may identify whether the two agents bind to the same site on the receptor. If each allosteric modulator binds to its own site, then the experimental data can be used to calculate their respective affinities, factors of cooperativity with tracer as well as their mutual cooperativity.

For the judgement whether two allosteric modulators bind to the same site mere comparison of experimental data with the theoretical binding curve calculated according to Eq 5 based on affinities and factors of cooperativity determined in separate experiments is sufficient. However, strong negative cooperativity between modulators A and B may be hard to differentiate from competition for the same site. In such case the apparent equilibrium dissociation constants of modulator A (K’_A_) at various high concentrations of allosteric modulator B should be examined. The value of K’_A_ is given either by Eq 9 when modulators A and B compete for the same site or by Eq 10 when each modulator binds to its own site. The principal difference at saturating concentrations of B is as follows: If A and B compete for the same site, an increase in concentration of B results in a linear increase in the K’_A_ value with slope equal to 1. If each of A and B bind to its own site, increasing the concentration of B makes K’_A_ slowly approach the limit value given by Eq 10.

Eq 6 includes 7 parameters that fully describe tracer binding at a fixed concentration in the presence of two allosteric modulators. For reliable fitting of this equation to the data as many parameters as possible must be fixed to the value determined in different sets of experiments. Specifically, K_X_ of tracer should be determined in saturation binding experiment. Values of K_A_, α, K_B_ and β should be determined in separate binding experiments with a fixed concentration of tracer by fitting Eq 3 (substituted in Eq 4) to the data. Yet with these 5 parameters fixed, the global fit of Eq 6 to several binding curves with various concentrations of modulator B may be necessary to estimate the values of γ and δ reliably. Alternatively, values of γ and δ can be determined by fitting Eq 10 to K’_A_ values for various combinations of the concentrations of tracer X and allosteric modulator B.

The same mathematics that describe the binding of two allosteric modulators each binding to its own site can be used to describe the binding of one allosteric modulator to two allosteric sites on the receptor (Eq 7). However, unlike the case of two allosteric modulators, the values of K_A1_, α_1_, K_A2_ and α_2_ cannot be determined in separate experiments in this scenario. Binding of an allosteric modulator to the low-affinity site is always affected by its binding to the high-affinity site. Thus, the estimation of binding parameters of such allosteric modulators is limited to their apparent values (e.g. K’_A_ value that is given by Eq 10). However, apparent binding parameters may be sufficient for many purposes, e.g. comparing relative apparent affinities of various allosteric modulators.

In summary, equilibrium binding studies of two allosteric modulators and a fixed concentration of tracer may provide valuable information about the nature of the interaction between these allosteric modulators. However, the exact determination of individual parameters of binding of two molecules of one allosteric modulator to two allosteric sites is possible only when the difference in affinity for high- and low- affinity binding sites is large.

## Supporting information

S1 Text Derivation of equations. (PDF)

S1 Zip archive Python code to simulate data and fit equations. (ZIP)
