# Supplementary material for: Analysis of equilibrium binding of an orthosteric tracer and two allosteric modulators
Source: PLoS One. 2019 Mar 27;14(3):e0214255. doi: 10.1371/journal.pone.0214255 (PMC6436737; doi:10.1371/journal.pone.0214255)
Supplement: S1 Text — (PDF) [file pone.0214255.s001.pdf]

Supplementary information: Analysis of equilibrium binding of the tracer and two allosteric modulators

Jan Jakubík, Alena Randáková, Esam E. El-Fakahany, and Vladimír Doležal

Derivation of equations

The derivation of the equations for tracer binding expressed as a ratio of the tracer binding in the presence of allosteric modulator (Y') to the tracer binding in the absence of allosteric modulator (Y) based on equilibrium dissociation constants (K) and factors of cooperativity (Greek letters) shown in main manuscript is described below. These equations are based on the Fig 2 and Fig 3 of the main manuscript describing interaction of tracer X and two allosteric modulators A and B. The relationship between the concentration of the ligands and their complexes is derived assuming that the reactions take place under pseudo-first order conditions when the ligands are present in sufficient excess over the receptor so that the formation of the complexes leaves the free concentration of ligands virtually unchanged.

Derivations for Fig 2 – Modulators A and B bind to the same site

Definitions:

$$K_X = \frac{[X][R]}{[XR]} \quad [A1]$$

$$K_A = \frac{[R][A]}{[RA]} \quad [A2]$$

$$K_B = \frac{[R][B]}{[RB]} \quad [A3]$$

$$\alpha K_X = \frac{[X][RA]}{[XRA]} \quad [A4]$$

$$\alpha K_A = \frac{[XR][A]}{[XRA]} \quad [A5]$$

$$\beta K_X = \frac{[X][RB]}{[XRB]} \quad [A6]$$

$$\beta K_B = \frac{[XR][B]}{[XRB]} \quad [A7]$$

Substitutions:

$$\frac{[XRA]}{[X][R]} = \frac{[A]}{\alpha K_X K_A} \quad [A8]$$

$$\frac{[XRB]}{[X][R]} = \frac{[B]}{\beta K_B K_X} \quad [A9]$$

$$\frac{[RA]}{[X][R]} = \frac{[A]}{K_A [X]} \quad [A10]$$

Tracer fractional occupancy in the absence of allosteric modulators

$$Y = \frac{[X][R_{TOT}]}{[X]+K_X} \quad [A11]$$

Total number of receptors

$$R_{TOT} = [R]+[XR]+[RA]+[RB]+[XRA]+[XRB] \quad [A12]$$

Tracer fractional occupancy in the presence of allosteric modulators

$$Y' = \frac{[XR]+[XRA]+[XRB]}{R_{TOT}} \quad [A13]$$

After substitution

$$Y' = \frac{\frac{[X]}{K_X}+\frac{[A][X]}{\alpha K_A K_X}+\frac{[B][X]}{\beta K_B K_X}}{1+\frac{[A]}{K_A}+\frac{[B]}{K_B}+\frac{[X]}{K_X}+\frac{[A][X]}{\alpha K_A K_X}+\frac{[B][X]}{\beta K_B K_X}} \quad [A14]$$

$$Y' = \frac{\frac{[X]}{K_X}(1+\frac{[A]}{\alpha K_A}+\frac{[B]}{\beta K_B})}{1+\frac{[A]}{K_A}+\frac{[B]}{K_B}+\frac{[X]}{K_X}(1+\frac{[A]}{\alpha K_A}+\frac{[B]}{\beta K_B})} \quad [A15]$$

$$\omega = 1+\frac{[A]}{\alpha K_A}+\frac{[B]}{\beta K_B} \quad [A16]$$

$$Y' = \frac{[X]\omega}{K_X(1+\frac{[A]}{K_A}+\frac{[B]}{K_B}+\frac{[X]}{K_X}\omega)} \quad [A17]$$

Eq. A17 divided by Eq. A11:

$$Y'/Y = \frac{([X]+K_X)\omega}{K_X(1+\frac{[A]}{K_A}+\frac{[B]}{K_B}+\frac{[X]}{K_X}\omega)} \quad [A18]$$

$$Y'/Y = \frac{([X]+K_X)\omega}{K_X+\frac{K_X[A]}{K_A}+\frac{K_X[B]}{K_B}+[X]\omega} \quad [A19]$$

$$Y'/Y = \frac{([X]+K_X)\omega}{K_X(1+\frac{[A]}{K_A}+\frac{[B]}{K_B})+[X]\omega} \quad [A20]$$

Using algebraic equality:

$$\frac{(x+k)\omega}{ka+x\omega} = \frac{x+k}{x+k\frac{a}{\omega}}$$

$$Y'/Y = \frac{[X]+K_X}{[X]+K_X\frac{1+\frac{[A]}{K_A}+\frac{[B]}{K_B}}{\omega}} \quad [A21]$$

$$Y'/Y = \frac{[X]+K_X}{[X]+K_X\frac{1+\frac{[A]}{K_A}+\frac{[B]}{K_B}}{1+\frac{[A]}{\alpha K_A}+\frac{[B]}{\beta K_B}}} \quad [A22]$$

Derivations for Fig 3 – Modulators A and B bind to two sites

Definitions in addition to Fig 2:

$$\gamma K_A = \frac{[RB][A]}{[RAB]} \quad [A23]$$

$$\gamma K_B = \frac{[RA][B]}{[RAB]} \quad [A24]$$

$$\alpha\beta\lambda\gamma K_X = \frac{[RAB][X]}{[XRAB]} \quad [A25]$$

$$\alpha\kappa\beta\gamma K_A = \frac{[XRB][A]}{[XRAB]} \quad [A26]$$

$$\zeta\alpha\beta\gamma K_B = \frac{[XRA][B]}{[XRAB]} \quad [A27]$$

$$\lambda\gamma = \kappa\beta = \zeta\alpha = \delta \quad [A28]$$

$$\delta\alpha\beta K_X = \frac{[RAB][X]}{[XRAB]} \quad [A29]$$

$$\delta\alpha\gamma K_A = \frac{[XRB][A]}{[XRAB]} \quad [A30]$$

$$\delta\beta\gamma K_B = \frac{[XRA][B]}{[XRAB]} \quad [A31]$$

Substitutions in addition to Fig 2:

$$\frac{[XRAB]}{[X][R]} = \frac{[A][B]}{\delta\alpha K_X\gamma K_A K_B} \quad [A32]$$

Total number of receptors

$$R_{TOT} = [R]+[XR]+[RA]+[RB]+...$$

$$...+[XRA]+[XRB]+[RAB]+[XRAB] \quad [A33]$$

Tracer fractional occupancy in the presence of allosteric modlators

$$Y' = \frac{[XR]+[XRA]+[XRB]+[XRAB]}{R_{TOT}} \quad [A34]$$

$$Y' = \frac{\frac{[X]}{K_X}+\frac{[A][X]}{\alpha K_A K_X}+\frac{[B][X]}{\beta K_B K_X}+\frac{[A][B][X]}{\delta\alpha K_X\gamma\beta K_A K_B}}{1+\frac{[A]}{K_A}+\frac{[B]}{K_B}+\frac{[A][B]}{\gamma K_A K_B}+\frac{[X]}{K_X}+\frac{[A][X]}{\alpha K_A K_X}+\frac{[B][X]}{K_B K_X}+\frac{[A][B][X]}{\alpha\gamma\delta K_A K_B K_X}} \quad [A35]$$

$$Y' = \frac{\frac{[X]}{K_X}(1+\frac{[A]}{\alpha K_A}+\frac{[B]}{\beta K_B}(1+\frac{[A]}{\alpha\gamma\delta K_A}))}{1+\frac{[A]}{K_A}+\frac{[B]}{K_B}(1+\frac{[A]}{\gamma K_A})+\frac{[X]}{K_X}(1+\frac{[A]}{\alpha K_A}+\frac{[B]}{K_B}(1+\frac{[A]}{\alpha\gamma\delta K_A}))} \quad [A36]$$

$$\psi = (1+\frac{[A]}{\alpha K_A}+\frac{[B]}{\beta K_B}(1+\frac{[A]}{\alpha\gamma\delta K_A})) \quad [A37]$$

$$Y' = \frac{[X]\psi}{K_X(1+\frac{[A]}{K_A}+\frac{[B]}{K_B}(1+\frac{[A]}{\gamma K_A})+\frac{[X]}{K_X}\psi)} \quad [A38]$$

Eq. 38 divided by Eq. A11:

$$Y'/Y = \frac{([X]+K_X)\psi}{K_X(1+\frac{[A]}{K_A}+\frac{[B]}{K_B}(1+\frac{[A]}{\gamma K_A})+\frac{[X]}{K_X}\psi)} \quad \text{[A39]}$$

$$Y'/Y = \frac{([X]+K_X)\psi}{K_X\frac{[A]}{K_A}+K_X\frac{[B]}{K_B}(1+\frac{[A]}{\gamma K_A})+[X]\psi} \quad \text{[A41]}$$

$$Y'/Y = = \frac{([X]+K_X)\psi}{K_X(1+\frac{[A]}{K_A}+\frac{[B]}{K_B}(1+\frac{[A]}{\gamma K_A}))+[X]\psi} \quad \text{[A42]}$$

Using algebraic equality:

$$\frac{(x+k)\psi}{ka+x\psi} = \frac{x+k}{x+k\frac{a}{\psi}}$$

$$Y'/Y = \frac{[X]+K_X}{[X]+K_X\frac{1+\frac{[A]}{K_A}+\frac{[B]}{K_B}(1+\frac{[A]}{\gamma K_A})}{\psi}} \quad \text{[A43]}$$

$$Y'/Y = \frac{[X]+K_X}{[X]+K_X\frac{1+\frac{[A]}{K_A}+\frac{[B]}{K_B}(1+\frac{[A]}{\gamma K_A})}{1+\frac{[A]}{\alpha K_A}+\frac{[B]}{\beta K_B}(1+\frac{[A]}{\alpha\gamma\delta K_A})}} \quad \text{[A44]}$$
